# Coronavirus disease 2019 in patients with rheumatic immune-mediated diseases in a single University Hospital, matched case-control study and literature review

**DOI:** 10.3389/fmed.2022.1056374

**Published:** 2022-12-12

**Authors:** David Martínez-López, Ivan Ferraz-Amaro, Diana Prieto-Peña, Lara Sánchez-Bilbao, Alba Herrero-Morant, Carmen Álvarez-Reguera, Fabricio Benavides-Villanueva, Cristina Corrales-Selaya, Martín Trigueros-Vázquez, Miguel Ángel González-Gay, Ricardo Blanco

**Affiliations:** ^1^Department of Rheumatology, Hospital Universitario Marqués de Valdecilla, IDIVAL, Santander, Spain; ^2^Department of Rheumatology, Hospital Universitario de Canarias, Santa Cruz de Tenerife, Spain

**Keywords:** COVID-19, autoimmune diseases, antirheumatic agents, rituximab, biologic agents

## Abstract

**Background:**

COVID-19 may present different degrees of severity. Viral infections in patients with rheumatic inflammatory diseases (R-IMID) trend to present more severe disease. However, data comparing the severity of the disease between R-IMID and the general population are scarce.

**Objectives:**

To compare predisposing factors, clinical, serological features, and severity of COVID-19 infection in patients with and without R-IMID.

**Methods:**

Case-control study in a single University Hospital. We included all consecutive patients with a diagnosis of an R-IMID and COVID-19 infection up to March 31st, 2021. This cohort was compared to patients without R-IMID and not receiving immunosuppressive therapy, matched for sex and age (±5 years). Confirmed infection was defined if a patient had a positive nasopharyngeal swab for SARS-CoV-2. Severity was divided into mild, moderate, severe and critical according to the United States National Institute of Health (NIH) guidelines.

**Results:**

We included 274 R-IMID patients (185 women/89 men), mean age 59.1 ± 18 years. More frequent R-IMID were: Rheumatoid arthritis (28.8%), Psoriatic Arthritis (20.1%), axial Spondyloarthritis (12.4%), Polymyalgia Rheumatica (8%) and Systemic Lupus Erythematosus (8%). Hypertension and dyslipidemia were more frequent in patients with R-IMID. Although most of the cases were mild, critical cases and deaths were more frequent in R-IMID. When adjusted by comorbidities, no statistical differences were observed.

**Conclusion:**

R-IMID have a very similar clinical presentation when compared to the general population. There is a trend to an increased severity of the disease in patients with R-IMID.

## Highlights

### What is already known about this topic

It is not clearly established whether patients with R-IMID are at risk of a more severe COVID-19. Regarding the treatments received, it is known that Rituximab is associated with a worse prognosis. This is unclear for other treatments used in R-IMID. Risk factors specific to patients with R-IMID have not been established.

### What this study adds

Patients with R-IMID present a trend to an increased severity of COVID-19 infection, not reaching statistical significance when adjusted by comorbidities. Receiving Rituximab is associated with worse outcomes in patients with COVID-19 and R-IMID. We identified various severity factors for severe disease in patients with COVID-19 and R-IMID. Of these factors, the one with higher odds ratio was receiving Rituximab.

### How this study might affect research, practice or policy

These findings might be useful for a better risk stratification for patients with COVID-19 and R-IMID. This stratification can be useful to decide which patients might benefit from treatments for COVID-19 at the onset of the disease.

## Introduction

Since the first cases described in Wuhan (China) in December 2019, coronavirus disease 2019 (COVID-19) caused by the severe acute respiratory syndrome coronavirus 2 (SARS-CoV-2) has become a global pandemic (1, 2).

According to the World Health Organization (WHO), there have been more than four hundred million cases of COVID-19, including almost four million deaths (2).

The severity of COVID-19 ranges from asymptomatic or mild cases to a life-threatening disease that can result in fatal outcomes. Several poor prognostic factors have been identified, including advanced age, obesity, smoking history, diabetes mellitus, and immunosuppression (3).

Patients with rheumatic immune-mediated inflammatory diseases (R-IMID) may have a global increased risk of infections due to the disease itself, comorbidities and/or immunosuppressive therapy (4–6).

However, data regarding the severity of COVID-19 in patients with R-IMID and immunosuppressive therapy have been controversial. Some studies have found an increased risk of COVID-19 severity in patients with R-IMID (7–10).

By contrast, other studies have not confirmed these results, and conclude that there is no increased risk of severity in patients with R-IMID (11–14).

However, most of the evidence mentioned relies on small case series of patients and seldom compares the results in these patients with the general population.

A recent systematic review and meta-analysis by Xu et al. (15) including 31 articles and 1,138 patients concluded that patients with R-IMID have substantial rates of severe outcomes. However, this study shows important geographical variations in these outcomes and most of the studies included do not establish a comparison with the general population.

Regarding immunosuppressive therapy received by R-IMID patients, the available evidence points to an increased severity of COVID-19 in patients in treatment with rituximab (RTX) (9, 16, 17).

However, data regarding other immunosuppressive therapies, such as glucocorticoids, cDMARDs or other biologic or synthetic DMARDs remain unclear (10, 14, 18, 19).

In the previous studies carried out in populations with COVID-19 and R-IMID, some comorbidities have been reported to be related with worse outcomes. This is the case of older age at presentation, hypertension, diabetes mellitus, obesity and previous heart disease (8, 19–22).

Lymphopenia has also been identified as a risk factor for severe COVID-19 in the general population. However, this has not been assessed in the population with R-IMID. High levels of CRP, D-dimer, LDH, ferritin, IL-6, and elevated liver enzymes and creatinine have also been proposed as increased severity factors (22).

To our knowledge, the difference between the clinical presentation of COVID-19 and the laboratory findings between patients with R-IMID and the general population have not been analyzed. In the general population, the presence of dyspnea and gastrointestinal symptoms have been associated with increased severity of the disease (22).

To sum up, most of the evidence published points toward an increased severity of COVID-19 in patients with R-IMID, but the evidence behind this statement is still weak Therefore, further research is needed on this topic. The purpose of our study was to compare predisposing factors, clinical, serological features, severity and mortality of COVID-19 infection in patients with and without R-IMID.

## Patients and methods

### Patients and study design

We carried out a retrospective case-control study in a single University Hospital. We included all consecutive patients with a diagnosis of a R-IMID and a positive test for COVID-19 up to March 31st, 2021.

Rheumatic immune-mediated inflammatory disease was diagnosed by expert rheumatologists from our center according to current classification criteria for each R-IMID. Confirmed COVID-19 was defined if the patient had a positive polymerase chain reaction (PCR) test in nasopharyngeal swab for SARS-CoV-2.

A total of 274 patients with R-IMID and COVID-19 infection were identified. Therefore, 274 controls were selected, and matched by sex and age (±5 years). Controls were defined as patients with a positive PCR in nasopharyngeal swab for SARS-CoV-2, but without any R-IMID diagnosis and not receiving immunosuppressive therapy.

A total of 70 patients of each group (25.5%) required hospitalization due to the severity of COVID-19. The rest of the patients of this study (74.5%) were not admitted to the hospital.

Many classifications have been used to classify the different severity grades of the disease. We used the United States National Institute of Health (NIH) guidelines since they are one of the most trusted. This guideline divides COVID-19 case severity into mild, moderate, severe or critical disease according to various clinical and radiological parameters (23).

### Outcome variables and clinical definitions

The main outcome variables for this study were: (a) Severity of COVID-19 in patients with R-IMID and controls according to NIH guidelines and (b) Mortality of COVID-19 in both groups.

Data were obtained from the patients’ clinical records after the patient was considered cured from the disease or suffered a mortal outcome. Patients were considered to be cured from COVID-19 when the symptoms disappeared and a negative PCR test in nasopharyngeal swab for SARS-CoV 2 was obtained.

For all patients with COVID-19, we obtained the following data from their medical records: Sex, age, presence of comorbidities and cardiovascular risk factors, previous diagnosis of R-IMID, treatment for R-IMID at the onset of the symptoms, previous therapies for R-IMID, COVID-19 symptoms, analytical data, including CRP, creatinine, leukocytes, lymphocytes (at the onset of COVID-19 and previous), hemoglobin, platelets, ferritin, LDH, D-Dimer…), therapies for COVID-19 and severity of the disease according to NIH guidelines and mortality.

### Statistical analysis

Results were expressed as mean ± standard deviation (SD) for variables with a normal distribution, or as median and interquartile range (IQR) for those not normally distributed. Percentages were shown for categorical variables Student’s *t*-test or Mann-Whitney U-test were used to compare continuous variables as appropriate. The chi-square test or the Fisher exact test were used to assess differences between categorical variables. Multivariable logistic regression analysis, adjusting for confounders, was assessed to analyze the association between COVID-19 infection severity and mortality. Confounding variables were selected from demographics and traditional CV risk factors if they had a *p-*value lower than 0.20. A *p*-value <0.05 was considered statistically significant in all the calculations. Data management and analysis were performed using SPSS for Windows version 25 (SPSS Inc, Chicago, IL, USA).

### Literature review

We carried out a review of the literature in search of previous case-control studies performed in patients with COVID-19 and R-IMID.

The search was performed in the month of December 2021. The objective of this review was clarifying if patients with R-IMID have a more severe course of COVID-19.

We searched articles in the bases of PubMed, Scielo, and Cochrane Library.

We used the terms COVID-19 + SARS-CoV2 + R-IMID + rheumatic inflammatory disease + rheumatoid arthritis + connective tissue disease + case-control in different combinations. The inclusion criteria were: (1) the study included both patients with R-IMID and COVID-19, and (2) the study was a case-control study.

## Results

We included 274 patients with R-IMID patients and COVID-19 infection. Table 1 summarizes the underlying diseases and immunosuppressive therapy. The most frequent R-IMID was rheumatoid arthritis (*n* = 79, 28.8%), followed by psoriatic arthritis (*n* = 55, 20.1%) and spondyloarthritis (*n* = 34, 12.4%). At the onset of COVID-19 infection, 27% of the patients (*n* = 75) were receiving treatment with at least one conventional DMARD. The most frequently used cDMARD was methotrexate (*n* = 62, 23%) followed by hydroxychloroquine (*n* = 50, 18%). Biological DMARDs were used in 60 patients (21.9%), being the most frequently used TNF inhibitors (*n* = 41, 15%). Also, a considerable number of patients (*n* = 77, 28%) were receiving treatment with oral glucocorticoids.

**TABLE 1:** Underlying diseases and immunosuppressive therapy in 274 patients with inflammatory rheumatology diseases complicated with coronavirus disease 2019 (COVID-19).

Variables	Patients (*n* = 274)
**Inflammatory rheumatic disease, *n* (%)** – Rheumatoid arthritis – Psoriatic arthritis – Spondyloarthritis – Polymyalgia rheumatica – Systemic lupus erythematosus – Vasculitis – Sjogren’s syndrome – Uveitis – Sarcoidosis – Behçet’s disease – Idiopathic inflammatory myopathies – Systemic sclerosis – Others	79 (28.8) 55 (20.1) 34 (12.4) 22 (8) 22 (8) 8 (2.9) 8 (2.9) 6 (2.2) 5 (1.8) 5 (1.8) 3 (1.1) 2 (0.7) 25 (9.1)
**Treatments at the onset of COVID-19, *n* (%)** – Prednisone – Prednisone dose (mg/day) – Conventional DMARDS – Methotrexate – Hydroxychloroquine – TNF inhibitors – Leflunomide – Anti-CD20 – Anti-IL17 – Sulfasalazine – Azathioprine – Baricitinib – Abatacept – Calcineurin inhibitors – Anti-IL1 – Tofactinib – Upadacitinib – Mycophenolate mophetil – Anti-IL6	77 (28.1) 6.3 (2.5–40) 75 (27.4) 62 (22.6) 50 (18.3) 41 (15) 16 (5.8) 8 (2.9) 8 (2.9) 6 (2.2) 6 (2.2) 4 (1.5) 3 (1.1) 2 (0.7) 2 (0.7) 2 (0.7) 1 (0.4) 1 (0.4) 0

We also included 274 COVID-19 matched-controls without R-IMID or immunosuppressive therapy. The main characteristics of both groups are shown in Table 2.

**TABLE 2:** Demographics, cardiovascular risk factors and comorbidities in coronavirus disease (COVID) patients with inflammatory rheumatology diseases versus controls.

Variables	Controls (*n* = 274)	Patients (*n* = 274)	*P*
Age (years)	59 ± 17	59 ± 18	0.86
Female, *n* (%)	185 (67)	185 (67)	–
**Cardiovascular risk factors, *n* (%)** – Hypertension – Dyslipidemia – Obesity – Diabetes mellitus – Current smoker	84 (30.7) 79 (28.8) 49 (17.9) 37 (13.5) 18 (6.6)	119 (43.4) 119 (43.4) 49 (17.9) 36 (13.1) 27 (9.9)	0.002* <0.001* – 0.9 0.13
**Comorbidities, *n* (%)** – Cardiovascular disease – Cancer – Chronic kidney disease – Chronic obstructive lung disease – Chronic liver disease – Inflammatory bowel disease – Interstitial lung disease	33 (12) 12 (4.4) 11 (4) 9 (3.3) 9 (3.3) 2 (0.7) 2 (0.7)	47 (17.2) 21 (7.7) 27 (9.9) 7 (2.6) 11 (4) 6 (2.2) 5 (1.8)	0.09 0.11 0.007* 0.62 0.65 0.29 0.45

*p < 0.05.

Bold values represent the statistically significant difference.

In both groups most patients were female (67%) with a mean age of 59 ± 18 years.

Hypertension, dyslipidemia and chronic kidney disease were significantly more frequent in patients with R-IMID when compared to controls. No other significant differences were found in the remaining comorbidities analyzed.

In the whole group, most of the patients (*n* = 383, 69.9%) showed symptoms related to COVID-19, while the remaining were asymptomatic (Table 3).

**TABLE 3:** Main clinical, analytical and therapy of coronavirus (COVID) disease in patients with rheumatic immune-mediated diseases (IMIDs) and case controls.

	Controls (*n* = 274)	Patients (*n* = 274)	*p*
**COVID symptoms**	194 (71)	189 (70)	0.89
Fever	109 (40)	122 (45)	0.79
Headache	52 (19)	30 (11)	0.41
Asthenia	55 (20)	61 (22)	0.83
Myalgia	47 (17)	44 (16)	0.28
Diarrhea	48 (18)	23 (8)	**<0**.**001**
Cough	92 (34)	128 (47)	0.55
Expectoration	29 (11)	21 (8)	86
Thorax pain	21 (8)	22 (8)	0.75
Odynophagia	54 (20)	33 (12)	**0**.**001**
Dyspnea	30 (11)	62 (23)	**0**.**003**
Ageusia	29 (11)	45 (16)	0.16
Anosmia	26 (9)	41 (15)	0.17
Skin lesions	1 (0)	4 (1)	0.65
**Laboratory data**			
CRP, mg/dl	3.9 (1.0–7.3)	4.1 (1.4–8.10)	0.59
Creatinine (mg/dl)	0.9 ± 0.4	1.1 ± 1.0	0.23
Platelets (x103/ul)	199 ± 89	206 ± 90	0.60
Hemoglobin (g/l)	14.0 ± 1.4	13.0 ± 1.8	**<0**.**001**
Total leukocytes (x103/ul)	–	–	–
Neutrophils (x103/ul)	4446 ± 2565	4510 ± 2554	0.87
Lymphocytes (x103/ul)	1182 ± 673	1112 ± 668	0.49
Previous lymphocytes (x103/ul)	2015 ± 725	1951 ± 852	0.47
Ferritin (ug/L)	615 ± 685	426 ± 417	0.067
LDH (U/L)	266 ± 74	257 ± 92	0.49
D-Dimer (ng/ml)	854 ± 839	1154 ± 1285	0.092
**Therapies during COVID infection**			
No treatment required	220 (80)	208 (76)	0.19
Remdesivir	4 (1)	1 (0)	0.22
Azithromycin	52 (19)	39 (14)	0.13
Lopinavir/ritonavir	24 (9)	19 (7)	0.42
Hydroxychloroquine	45 (16)	36 (13)	0.27
Anti-IL1	2 (1)	2 (1)	0.99
Anti-IL6	6 (2)	2 (1)	0.18
Intravenous glucocorticoids	21 (8)	30 (11)	0.19
Plasma therapies	9 (3)	9 (3)	0.99
**Hospitalization data**			
Hospitalization *n*, (%)	70 (26)	70 (26)	–
Presence of pneumonia in chest X-ray *n*, (%)	63 (23)	56 (20)	0.47
Admission to ICU, *n* (%)	5 (2)	10 (4)	0.19

Data represent average ±SD or median (interquartile range). Bold values represent the statistically significant difference.

The most frequent symptoms were: fever (*n* = 231, 42.2%), cough (*n* = 220, 40.1%) and asthenia (*n* = 116, 21.2%). Dyspnea was more frequent in the R-IMID group, while diarrhea and odynophagia were more frequent in the control group. No other statistically significant differences were found in the rest of the symptoms between both groups.

When analyzing laboratory parameters (Table 3) no differences were found between both groups in the values of C-reactive protein, platelets, leukocytes, lymphocytes, ferritin or D-Dimer. Hemoglobin values were significantly lower in the R-IMID group.

Therapies used for COVID-19 in both groups are shown in Table 3.

Other data regarding the severity of the disease are shown in Table 3. As mentioned previously, 70 patients (25.5%) in each group were admitted to the hospital. Therefore, there were no differences regarding the hospitalization rate between both groups. Presence of pneumonia in plain chest X-ray was found in 63 patients (23%) in the control group, and in 56 patients (20%) in the R-IMID group. No statistical differences were found between both groups.

A total of five patients (2%) in the control group were admitted to the intensive care unit (ICU), while 10 patients (4%) in the R-IMID were admitted to the ICU. However, these differences did not reach statistical significance.

Statistical analysis of severity of patients with R-IMID compared to controls is shown in Table 4. In the crude analysis the results indicate that there is a significant difference between both groups with an increased severity in patients with R-IMID.

**TABLE 4:** Coronavirus disease 2019 (COVID-19) severity and deaths in patients and controls.

	Controls (*n* = 274)	Patients (*n* = 274)	*P*	Crude OR (95% CI)	*p*	*Adjusted OR (95% CI)	*p*
**Severity**							
Mild	204 (74)	209 (76)	**0.031**	–	–	–	–
Moderate	47 (17)	35 (13)		0.73 (0.45–1.17)	0.19	**0.49 (0.29**–**0.83)**	**0.008**
Severe	14 (5)	8 (3)		0.56 (0.23-1.36)	0.56	0.48 (0.16-1.41)	0.18
Critical	9 (3)	22 (8)		**2.39 (1.07**–**5.31)**	**0.033**	1.31 (0.56–3.05)	0.54
Deaths	7 (3)	18 (7)	**0.024**	2.68 (1.10–6.53)	0.29	1.54 (0.60–3.98)	0.37

*Adjusted for hypertension, dyslipidemia, established cardiovascular (CV) disease, cancer, chronic kidney disease, NSAIDs use. OR, odds ratios represents the risk of death in patients compared to controls (reference variable); and the risk of being a patient as COVID-19 infection severity increases (mild severity reference variable). Bold values represent the statistically significant difference.

However, when these results are adjusted by comorbidities, there were no statistical significant difference between both groups.

When analyzing thrombotic events, three patients in the R-IMID group suffered thrombotic events (deep vein thrombosis in one case and pulmonary embolism in two cases) while only one patients in the control group suffered a thrombotic event (deep vein thrombosis). There were no statistically significant differences between both groups.

Regarding mortality, there were 18 deaths (7%) in the R-IMID group, with only seven deaths (3%) in the control group, with a crude odds ratio of 2.68. However, in the adjusted analysis, there were no statistically significant differences between both groups.

In Table 5, we compared the main characteristics of R-IMID patients who had mild COVID-19 infection with those who developed severe COVID-19 infection. In this analysis, we identified risk factors for severe COVID-19 infection in R-IMID patients: age higher than 65 years old, hypertension, dyslipidemia and the presence of cardiovascular, kidney or pulmonary diseases. Regarding analytical findings, the presence of increased levels of creatinine and lymphopenia were also associated with worse outcomes. Rituximab was the only treatment associated with severe disease.

**TABLE 5:** Clinical severity of 274 with rheumatic immune-mediated diseases (R-IMID) diagnosed with coronavirus disease 2019 (COVID-19) [analytical findings and Immunosuppressants are at coronavirus (COVID) diagnosis].

	Overall (*N* = 274)	Mild/Moderate (*N* = 245)	Severe/Critical (*N* = 29)	Mild/Moderate vs. Severe/Critical *P*
**Comorbidities (*n*, %)**				
Hypertension	119 (43.4)	96 (39.2)	23 (79.3)	0.0001*
Dyslipidemia	119 (43.4)	100 (40.8)	19 (65.5)	0.02*
Age higher than 65 years	100 (36.5)	75 (30.6)	25 (86.2)	0.0001*
Obesity	49 (17.9)	42 (17.1)	7 (24.1)	0.5
Diabetes mellitus	36 (13.1)	29 (11.8)	7 (24.1)	0.1
Pulmonary diseases	29 (10.6)	17 (6.9)	12 (41.4)	0.0001*
Cardiovascular diseases	45 (16.4)	34 (13.9)	11 (37.9)	0.002*
Chronic kidney disease	27 (9.9)	18 (7.3)	9 (31)	0.0002*
**Analytical findings (median ± IQR)**				
Creatinine (mg/dl)	0.91 ± 0.4	0.89 ± 0.38	1.31 ± 0.93	0.005*
Platelets (x103/μl)	179 ± 78	205 ± 91	140 ± 152	0.379
Lymphocytes (x103/μl)	1 ± 1	1.2 ± 0.5	0.6 ± 0.4	0.007*
D-Dimer (ng/ml)	999 ± 1256	701 ± 753	1333 ± 1578	0.110
**Immunosuppressants, *n* (%)**				
Oral GC	77 (28.1)	71 (29)	6 (20.7)	0.47
Mean prednisone dose (mg/day ± SD)	6.3 ± 6.3	6.1 ± 5.8	10 ± 11	0.66
HCQ	50 (18.2)	47 (19.2)	3 (10.3)	0.36
MTX/other cDMARDs	62/23 (22.6/8.4)	58/21 (23.7/8.6)	4/2 (13.8/6.9)	0.33/0.96
AZA	6 (2.2)	6 (2.4)	0	0.88
MMF	1 (0.4)	1 (0.4)	0	0.74
TNF inhibitors	31 (11.3)	30 (12.2)	1 (3.4)	0.29
RTX	8 (2.9)	4 (1.6)	4 (13.8)	0.002*
Other bDMARDs	19 (6.9)	16 (6.5)	3 (10.3)	0.71
JAKINIB	6 (2.2)	5 (2)	1 (3.4)	0.86

AZA, azathioprine; GC, glucocorticoids; HCQ, hydroxychloroquine; MM, mycophenolate mofetil; MTX, methotrexate; RTX, rituximab.

*P < 0.05.

Bold values represent the statistically significant difference.

**FIGURE 1:**
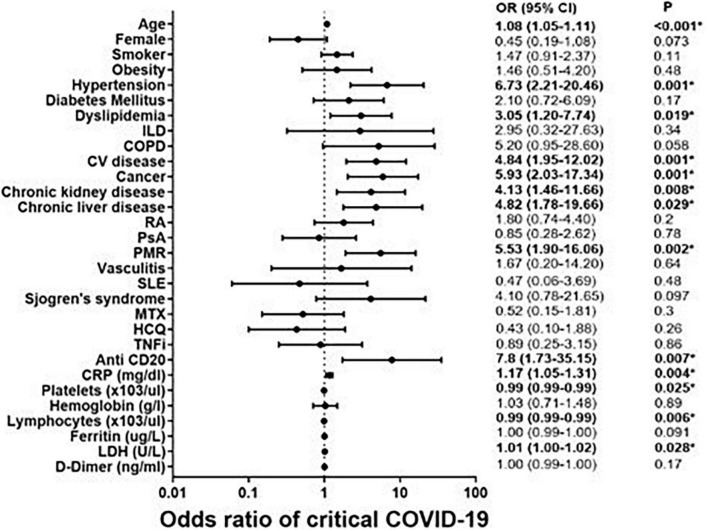
Predictive severity coronavirus disease 2019 (COVID-19) factors in 274 patients with rheumatic immune-mediated diseases (R-IMID).

These severity factors are also analyzed in Figure 1, in which the odds ratio for each factor has been calculated. Although the confidence intervals are wide due to the small sample, the largest OR were found in: treatment with anti-CD20 agents [OR = 7.8 (1.73–35.2)], the presence of hypertension [OR = 6.7 (2.2–20.5)] and previous cancer history [OR = 5.93 (2–17.3)].

### Literature review

In the search for literature review we only obtained 3 case-control studies that met both the inclusion, shown in Table 6. The details of these studies are shown in discussion.

**TABLE 6:** Literature review of previous case-control studies in patients with rheumatic immune-mediated diseases (R-IMID) and coronavirus disease 2019 (COVID-19).

References	Country	Number of patients with R-IMID and COVID-19	R-IMID included	R-IMID and severity of the disease	Immunosuppressive therapies and severity of the disease	Limitations
(24)	Italy	117	RA, PsA, SpA, PMR, vasculitis, SLE, systemic sclerosis, AOSD	Increased severity of COVID-19 in patients with R-IMID.	No difference between the different immunosuppressive therapies were found.	Older age and higher rate of comorbidities in patients with R-IMID
(8)	Spain	228	RA, SpA, PsA, and CTD.	Significantly greater risk of poor outcomes among hospitalized patients with CTD, but not in patients with inflammatory arthritis.	Immunosuppressive therapies were not associated with poor outcomes.	Only includes hospitalized patients.
(26)	Spain	78	RA, PsA, SpA, JIA, other (not specified)	Patients with R-IMID do not have an increased risk of hospitalization and mortality related to COVID-19.	Immunosuppressive therapies were not associated with increased hospitalization or mortality	Small sample size.
(27)	Spain	274	RA, PsA, SpA, PMR, vasculitis, Sjogren, SLE, systemic sclerosis, Behçet, inflammatory myopathies, sarcoidosis, ocular inflammatory diseases	Non-statistically significant trend toward more severe disease in patients with R-IMID when adjusted by cardiovascular risk factors.	Increased severity in patients receiving rituximab.	–

CTD, connective tissue diseases; JIA, Juvenile idiopathic arthritis; PMR, polymyalgia rheumatica; PsA, psoriatic arthritis; RA: rheumatoid arthritis; SLE, systemic lupus erythematosus; SpA, Axial spondyloarthritis.

## Discussion

Although many studies with patients with R-IMID and COVID-19 have been published, most of these studies were only case series and did not establish a comparison with the general population. We report the largest case-control study including 274 patients with R-IMID with COVID-19 infection. These data were compared with 274 controls matched by age and sex, with no diagnosis of R-IMID.

When analyzing these results and comparing patients with R-IMID to the patients in the control group, we found that there is greater severity in patients with R-IMID with COVID-19 according to the NIH scale of severity. However, when we adjust these results according to comorbidities, there are no significant differences, leaving only a slight trend toward greater severity. Therefore, greater severity of COVID-19 in our group of patients with R-IMID was probably due to the high number of comorbidities, and not an increased risk due to the disease itself. Regardless, it must also be taken into account that these comorbidities are more frequent in patients with some of these R-IMID. This has been clearly established, for example, in rheumatoid arthritis (24). RTX was identified as the only immunosuppressive therapy related to an increased severity of COVID-19 infection.

To our knowledge, only three previous case-control studies have been published (Table 6). The first case-control study involving patients with R-IMID and COVID-19 was published by Fredi et al. (1, 25). This study included 117 patients and 117 matched controls. This study concludes that there was an increased severity of COVID-19 in patients with R-IMID. However, patients with R-IMID were significantly older and with more comorbidities when compared to controls (25).

The second case-control study was performed by Pablos et al. (8). This multicenter study included only hospitalized patients. This study also concluded that there was an increased risk of poor outcomes in patients with connective tissue diseases. However, these poor outcomes were not observed in patients with inflammatory arthritis.

In contrast with the two previous studies, the study published by Mena et al. (26), found no increased severity (measured by risk of hospitalization and death) in patients with R-IMID. This study has a smaller sample size when compared to the previous (*n* = 78).

Our study includes the largest sample size when compared to the previous case-control studies published. In the previous studies, with the exception of Mena’s study (26), the higher prevalence of comorbidities and older age in patients with R-IMID might influence the results. In order to avoid potential confusion factors, we adjusted our results by age, sex, and comorbidities. Furthermore, in our study the severity was measured in a standardized and internationally accepted scale (NIH severity classification). The previous studies have used various methods to measure severity (necessity of hospitalization, mechanical ventilation, mortality…). However, measuring the outcomes makes it difficult to compare different studies and increases the variability of the results between centers.

In addition, we assessed for the first time the possible differences in the clinical presentation and laboratory parameters between R-IMID patients and controls. Our study shows that the clinical presentation in patients with R-IMID and the general population is very similar. With the exception of diarrhea and odynophagia which were more frequent in the controls, and dyspnea and cough, that were more frequent in the patients with R-IMID. Hemoglobin values were significantly lower in the R-IMID group. No other significant differences were found in laboratory parameters between the two groups.

Regarding the use of immunosuppressive therapy, none of the previous studies found correlation between any therapy and worse outcomes.

However, our study shows an increased severity of the disease in patients receiving rituximab, in line with the previous evidence obtained from other studies. (9, 16, 17).

Regarding treatments received for COVID-19, both groups received similar treatments, as shown in Table 3. However, treatment was constantly changing as scientific knowledge on COVID-19 evolved, so it’s difficult to assess the impact of these treatments in outcomes of our patients. In any case, most patients had mild disease and did not require any treatment.

Regardless, our study had some limitations and results must be carefully interpreted.

First, we would like to highlight that this study has some limitations due to its retrospective nature.

A second limitation, is that these cases were collected over a long period of time. During this time period, there was a rapid development of scientific knowledge around COVID-19. This translated into changes in treatment and better outcomes in these patients as time passed.

The last limitation is due to the heterogeneity of the diseases included. This limits the extrapolation of results to patients with a specific diagnosis.

## Conclusion

In conclusion, patients with R-IMID have a very similar clinical presentation when compared to the general population. Although there is a trend to an increased severity of the disease in patients with R-IMID, this trend did not reach statistical significance. Although our study shows some advantages over the previous ones, there are also some limitations due to its retrospective nature. Therefore, more research is needed to characterize the clinical features, risk factors and severity of COVID-19 infection in R-IMID infection.

## Patient and public involvement

Patients were first involved in this research at the start of the COVID-19 pandemic. Many patients consulted us with doubts about the new virus, how their disease could affect their prognosis, and if they were at a greater risk of presenting a worse outcome due to the medications they needed to treat their rheumatological disease. By then, no answers could be provided due to the lack of evidence. Therefore, we decided to launch this project to help the scientific community provide these answers. Therefore, for the design of this study we first took into account the concerns of our patients, paying special attention to the mortality caused by the disease. We have contact with various patient’s associations that will help us disseminate the results of this research. Also, some preliminary results have been announced in local newspapers.

## Data availability statement

The raw data supporting the conclusions of this article will be made available by the authors, without undue reservation.

## Ethics statement

The study protocol was approved by the Clinical Research Ethics Committee of the Hospital Marqués de Valdecilla (Santander, Spain) (Protocol number: 2020.151). Written informed consent from the patients/participants or patients/participants legal guardian/next of kin was not required to participate in this study in accordance with the national legislation and the institutional requirements.

## Author contributions

DM-L collected patient data by reviewed medical records, statistical analysis, and drafted the manuscript including tables and figures. IF-A performed advanced statistical analysis, elaboration of tables and figures, and reviewed the manuscript. DP-P prepared the patient database and reviewed the article. LS-B, AH-M, CÁ-R, FB-V, CC-S, and MT-V collected patient data by reviewed medical records. MG-G reviewed the article and elaborated the cover letter. RB provided the original idea, reviewed the article, and elaborated tables and figures. All authors contributed to the article and approved the submitted version.
